# Membrane Permeabilization by Pore-Forming RTX Toxins: What Kind of Lesions Do These Toxins Form?

**DOI:** 10.3390/toxins11060354

**Published:** 2019-06-18

**Authors:** Helena Ostolaza, David González-Bullón, Kepa B. Uribe, Cesar Martín, Jone Amuategi, Xabier Fernandez-Martínez

**Affiliations:** Departamento de Bioquímica y Biología Molecular (UPV/EHU) and Instituto Biofisika (UPV/EHU, CSIC), Aptdo. 644, 48080 Bilbao, Spain; david_go88@hotmail.com (D.G.-B.); kepa.belloso@ehu.eus (K.B.U.); cesar.martin@ehu.eus (C.M.); joneamuategi@gmail.com (J.A.); xfernandez016@gmail.com (X.F.-M.)

**Keywords:** pore-forming proteins, Gram-negative bacteria, bacterial protein toxins, RTX toxins, toroidal pores

## Abstract

Pore-forming toxins (PFTs) form nanoscale pores across target membranes causing cell death. The pore-forming cytolysins of the RTX (repeats in toxin) family belong to a steadily increasing family of proteins characterized by having in their primary sequences a number of glycine- and aspartate-rich nonapeptide repeats. They are secreted by a variety of Gram-negative bacteria and form ion-permeable pores in several cell types, such as immune cells, epithelial cells, or erythrocytes. Pore-formation by RTX-toxins leads to the dissipation of ionic gradients and membrane potential across the cytoplasmic membrane of target cells, which results in cell death. The pores formed in lipid bilayers by the RTX-toxins share some common properties such as cation selectivity and voltage-dependence. Hemolytic and cytolytic RTX-toxins are important virulence factors in the pathogenesis of the producing bacteria. And hence, understanding the function of these proteins at the molecular level is critical to elucidating their role in disease processes. In this review we summarize the current state of knowledge on pore-formation by RTX toxins, and include recent results from our own laboratory regarding the pore-forming activity of adenylate cyclase toxin (ACT or CyaA), a large protein toxin secreted by *Bordetella pertussis*, the bacterium causative of whooping cough.

## 1. Introduction

### Pore-Forming Proteins

**Figure 1:**
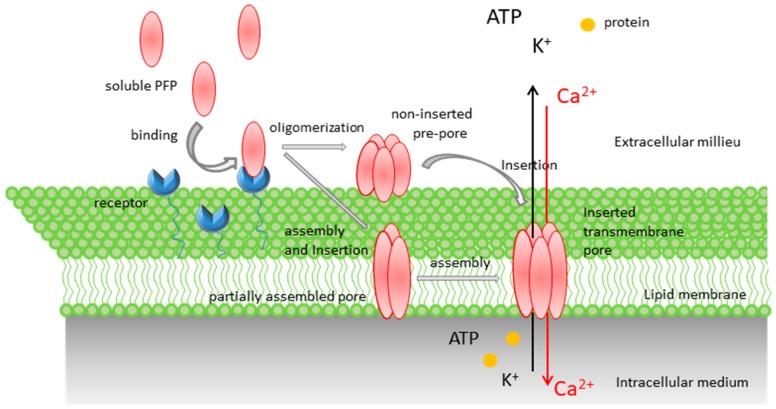
Simplified representation of the pore formation pathway of pore-forming proteins. Soluble pore-forming proteins (PFPs) are recruited to the host membrane by protein receptors and/or specific interactions with lipids. Upon membrane binding, toxins concentrate and start the oligomerization process, which usually follows one of two pathways. In the pathway followed by most β-PFPs, oligomerization occurs at the membrane surface, producing an intermediate structure known as a pre-pore (mechanism 1), which eventually undergoes conformational rearrangements that lead to concerted membrane insertion. In the pathway followed by most α-PFPs, PFP insertion into the membrane occurs concomitantly with a sequential oligomerization mechanism, which can lead to the formation of either a partially formed, but active, pore (mechanism 2), or the formation of complete pores. In both α-PFP and β-PFP pathways, the final result is the formation of a transmembrane pore with different architecture, stoichiometry, size, and conduction features, which promote the influx or efflux of ions, small molecules, and proteins through the host membrane.

Proteins that share the ability of creating hydrophilic “holes” that allow the passage of solutes (water, ions, or other biomolecules) through a wide variety of target membranes are called pore-forming proteins (PFPs); they are found in all kingdoms of life, from bacteria to humans [1,2,3]. PFPs are produced by their originating organisms usually as water-soluble units (generally monomers, dimers in some cases) and bind the target cell through sugars, lipids, or proteins present in the membrane that act as specific receptors. This binding step entails a reduction in the dimensions of protein diffusion from a three-dimensional volume to a two-dimensional plane, which leads to the increase in local concentration of the PFPs, and this favors oligomerization. Monomer assembly, a necessary step for pore formation in all known types of PFPs, can be anterior or concomitant to the exposure of hydrophobic protein surfaces that ultimately leads to membrane insertion (Figure 1). The identity of the PFP and its concentration around the target cell determine the mechanism and amplitude of the membrane permeabilization inflicted by the pores formed by PFPs. In turn, these differences may activate a large variety of cellular responses to PFPs and potential membrane repair pathways [4]. The most fatal outcome is cell death by necrosis, necroptosis, pyroptosis, or apoptosis.

PFPs have been traditionally classified into two main classes, α-PFPs and β-PFPs, on the basis of the secondary structure of the regions that penetrate the host cell membrane and are involved in the pore structure (α-helix or β-sheet, respectively) [1,5,6]. Due to their small hydrophobic insertion segment, β-PFPs need to oligomerize for insertion and spanning the lipid bilayer. To form the pore each of the monomers contributes with one or two β-strands that span back and forth across the lipid membrane, and create a transmembrane β-barrel with cylindrical structure. This specific configuration of the β-strands, where all hydrogen bonds are satisfied by inter-chain interactions, explains the higher structural stability of the β-pores relative to the α-pores, and why they have been better characterized. The majority of PFPs produced by bacteria are β-PFPs, the most studied group. Examples of β-PFPs are the *Staphylococcus aureus* α-toxin, the aerolysin family, the family of cholesterol-binding cytolysins, or the protective antigen (PA) of the anthrax toxin [7,8,9,10]. Pore formation by α-PFPs involves a structural change in the protein in which initially buried hydrophobic or amphipathic helical segments are exposed to the aqueous environment and as consequence such α-helical segments insert into the membrane. These prerequisites allow some α-PFPs to penetrate the membrane even in their monomeric form, which results in a molecular organization that may involve lipids and that is, in general, less defined and more dynamic than β-pores. Likely, because of this, the oligomerization mechanism of these PFPs has remained elusive for a long time, and for many α-PFPs it is not easy to separate oligomerization from pore formation [11]. Examples of α-PFPs are the actinoporins from sea anemones, colicins and cytolysin A (ClyA), both from *Escherichia coli*, the apoptotic Bcl-2 protein Bax, the diphtheria toxin from *Corynebacterium diphtheria*, or exotoxin A from *Pseudomonas aeruginosa*. Pore-forming toxins of the repeat in toxins (RTX) group are usually also considered members of this class.

The growing characterization of the structure and function of many PFTs in the last decades has enabled the development of biotechnological applications that target and engineer these proteins. Many pore-forming toxins have been proposed to be used as sensors for single molecule detection and characterization, including DNA and peptide sequencing. For example *Staphylococcus aureus* α-hemolysin, *Aeromonas hydrophila* aerolysin, or lysenin produced by the earthworm *Eisenia fetida*, among others, were reported for their potential use as single molecule sensors [12,13,14]. Such investigations that started in the 1990s by using pore-forming toxins as sensing elements have ultimately led to the development of a new, distinct, and highly relevant subfield in nanobiotechnology.

In the next sections below we discuss the current state of knowledge on a particular family of pore-forming proteins, namely, the RTX pore-forming toxin family, which is as large as it is unknown in many aspects. We also summarize recent information from our own laboratory on pore formation by one such RTX toxin, namely, the adenylate cyclase toxin (ACT or CyaA) secreted by *Bordetella pertussis*.

## 2. RTX Protein Family

**Figure 2:**
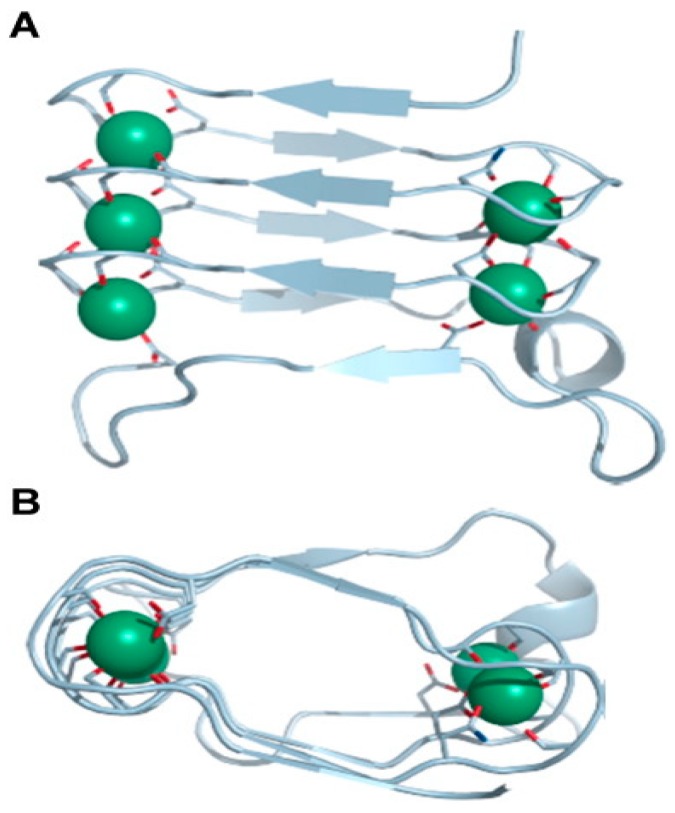
3D representation of the structure of part of the calcium binding β-roll motif of the alkaline protease from *Pseudomonas aeruginosa*. (**A**) Enlarged 3D view of the front face of the β-roll motif of alkaline protease (amino acids 328–400) including five bound Ca^2+^ ions (green spheres) (modified from PDB ID: 1KAP.pdb) [16]. Successive β strands within the ß-roll motif wind in a right-handed spiral. Ca^2+^ ions are bound within the turns between two strands by a repeated GGXGXD sequence motif (where X is any amino acid), meaning that each nonapeptide motif forms two half-sites for calcium ion binding between two turns. (**B**) 3D view looking down the axis of the β-roll including the five calcium ions (green spheres), the parallel β-strands, and tight turns around the metal-binding sites.

The so-called repeat in toxin (RTX) proteins constitute a superfamily of proteins exhibiting a wide range of biological activities and molecular masses ranging from 40 to over 600 kDa. It was Rodney A. Welch who in 1991 coined the name of the RTX family [15] to refer to a series of proteins characterized by the presence of arrays of glycine- and aspartate-rich nonapetide repeats in the C-terminal half of their respective amino acid sequences and extracellular secretion by the type I secretion systems (T1SS). The repeats contain the common sequence structure G–G–X–G–(N/D) –D–X–(L/I/V/W/Y/F)–X (where X can be any amino acid), but the number of repeats varies within RTX protein family members. These repeats form a parallel β-roll motif involved in calcium binding [16]. The successive beta strands within the β-roll motif wind in a right-handed spiral [16]. Ca^2+^ ions are bound within the turns between two strands by a repeated GGXGXD sequence (where X is any amino acid), which means that each nonapeptide motif forms two half-sites for calcium ion binding between two turns [16] (Figure 2).

The first described group of RTX proteins consists of toxins, mostly exhibiting a cytotoxic–cytolytic pore-forming activity, often first detected as a hemolytic halo surrounding bacterial colonies grown on blood agar plates [17,18,19,20]. Though for historical reasons the word “toxin” has been maintained in the family name, application of several types of screenings on sequenced bacterial genomes has allowed detection of over 1000 RTX family members with the biological functions of most of them remaining to be characterized [21]. Here we will circumscribe the family to the RTX toxins with pore-forming activity. We will discuss an overall view of pore formation and pore characteristics of these notable virulence factors, focusing on a particular toxin, the adenylate cyclase toxin (ACT or CyaA) secreted by *Bordetella pertussis*.

### 2.1. Pore-Forming RTX Toxins: Still Little-Known Orphans

The pore-forming RTX-toxin subfamily is a large family of Gram-negative bacterial pore-forming exotoxins. Toxins of the RTX family are encoded within the *rtx* operon. The general *rtx* gene cluster encodes three protein types: the RTX toxin (RtxA), an RTX toxin activating acyltransferase (RtxC), and dedicated T1SS proteins (RtxB, RtxD, and RtxE in some cases). The RTX toxin is synthesized as inactive precursor (pro-toxin) and then post-translationally modified by the cis-encoded RTX toxin activator. The RTX-activating acyltransferase catalyzes the covalent attachment of two fatty acids to conserved lysine residues within the RTX toxin. This modification is required in all pore-forming RTX toxins; however, its exact function in RTX toxicity is not fully understood. Toxin secretion from the producing bacteria occurs through an oligomeric secretion channel spanning across the entire Gram-negative bacterial cell envelope (i.e., cytoplasmic membrane, periplasmic space, and outer membrane). This dedicated ATP-binding cassette (ABC) transporter-based secretion apparatus recognizes a C-terminal uncleavable secretion signal and mediates a single-step translocation of the RTX polypeptides from the bacterial cytosol across both the inner and the outer bacterial membrane, directly into the extracellular space and without a periplasmic secretion intermediate (see reference [22] for a recent review). Pore-forming RTX toxins are produced by a broad range of Gram-negative pathogens including the genera *Escherichia*, *Bordetella*, *Proteus*, *Morganella*, *Moraxella*, and members of the *Pasteurellaceae* family (*Mannheimia*, *Pasteurella*, and *Aggregatibacter*) [21]. Among the RTX cytotoxins there are two groups, the conventional pore-forming leukotoxins and the more recently discovered very large multifunctional-autoprocessing repeats-in-toxin (MARTX) [23].

Traditionally RTX toxins have been divided into “hemolysins” and “leukotoxins”. The RTX “hemolysins” were initially found to exhibit broad target cell and species specificity, while the activity of “leukotoxins” was considered to be species and cell-type specific [20,24]. *E. coli* α-hemolysin (HlyA) for example shows detectable cytotoxic activity on a wide variety of cells from various species, including erythrocytes, granulocytes, monocytes, endothelial cells, or renal epithelial cells from mice, ruminants, and primates [25,26,27,28,29,30]. In contrast, *A. actinomycetemcomitans* (LtxA) and *M. haemolytica* (LktA) leukotoxins appear to be quite selective and cytotoxic only to a certain cell types in a species-specific manner [31,32,33]. More recently it was found that some of the so-called “hemolysins” such *E. coli* α-hemolysin (HlyA) or *Bordetella* adenylate cyclase toxin (ACT or CyaA) bind preferentially to leukocytes expressing the β_2_-integrins LFA-1 and Mac-1, respectively [34,35]. And so, it appears now more appropriate to consider that all pore-forming RTX toxins are primarily leukotoxins with different host specificities [21,36].

The pore-forming RTX-toxins are amphipathic polypeptides that have water-soluble precursors, but can also form membrane-spanning structures. This means that the molecules likely undergo substantial structural changes upon membrane binding. Interaction of the RTX toxins with the target cell membrane devoid of a specific proteinaceous receptor appears to occur in two steps, starting with a reversible adsorption of the toxin that is sensitive to electrostatic forces, which is then followed by an irreversible membrane insertion [37,38]. Adsorption of RTX toxins is detectable on both toxin-sensitive cells and on certain toxin-resistant cells [39,40]. Studies with the isolated calcium-binding domain of HlyA revealed that this part of the protein may adsorb on the membrane in the early stages of HlyA–membrane interaction [41].

Essential for hemolytic/cytolytic and cytotoxic activity of these RTX-toxins is the so-called hydrophobic domain, located near the toxin’s N-terminal end, which contains several hydrophobic and amphiphatic segments that are believed to potentially fold into α-helical structures capable of inserting into lipid bilayers and of forming hydrophilic pores. Mutations in the corresponding hydrophobic domains of several RTX toxins demonstrated that this N-terminal domain is critical for their ability to form transmembrane pores [42,43,44,45]. For *E. coli* HlyA several studies showed that the hydrophobic region was responsible for the insertion of the toxin into the target membrane [46,47], and recently it has been reported that its channel-forming domain might contain β-strands, in addition to α-helical structures [48]. In this regard, it has been quite surprising that in the recent publication by Brown et al. [49] they suggest that *Aggregatibacter actinomycetemcomitans* leukotoxin (LtxA) adopts a U-shaped conformation in the membrane, with the hydrophobic N-terminal and the C-terminal (secretion signal) domains residing outside of the membrane. It is thus clear that much remains to be known about the structure of this important domain. At this regard, it is striking that despite their relevance in the understanding of host/pathogen interactions, and the great advances in the last thirty years in our knowledge on the 3D-structure of many PFPs, the information about the RTX pore-forming toxins remains scarce. Many aspects, such as the structural characteristics of the pores formed in membranes, their possible stoichiometry, or the mechanistic details of pore assembly, insertion, etc., remain enigmatic at molecular level, constituting a long-standing gap in the field.

#### 2.1.1. Pore Formation by RTX Toxins

That RTX toxins form transmembrane pores in membranes was firstly suggested in 1986 by Bhakdi et al. [50] from hemolysis assays with the *Escherichia coli* HlyA hemolysin. Shortly after, formation of ion permeable pores was directly demonstrated for this same toxin using planar lipid membranes and conductance measurements [51,52]. Pore formation by HlyA was also determined in human macrophages using the patch clamp technique [53]. From then on, in less than a decade pore formation was reported for many other RTX toxins, including the *Actinobacillus pleuropneumoniae* hemolysin [54], the *Pasteurella haemolytica* leukotoxin [55], the adenylate cyclase toxin from *Bordetella pertussis* [56,57], the *Proteus vulgaris* and *Morganella morganii* hemolysins [58], the enterohemorragic *Escherichia coli* (EHEC) O157:H7 hemolysin [59], the *Actinobacillus pleurpneumoniae* ApxI, ApxII, and ApxII hemolysins [60], the *Actinobacillus actinomycetemcomitans* LtxA leukotoxin [61,62], and more recently pore formation by the *Kingella kingae* RtxA toxin has been noted [63]. From those biophysical studies several features were deducted for the RTX toxins pores: an apparently defined size in the range of ≈1–3 nm, with the apparent exception of the *Bordetella pertussis* adenylate cyclase toxin for which a size of 0.6–0.8 nm was proposed [56,64], cation-selectivity, short mean lifetimes and transient character, showing rapid fluctuations between open/closed states or different ion-conducting states, single-channel conductance values ranging from ≈300 to 1600 pS in 1M KCl, with the exception of the adenylate cyclase toxin with a conductance of 27 pS, and voltage-dependence.

A debated aspect regarding pore formation by the RTX toxins remains whether toxin oligomerization is or not involved, and consequently the possible real stoichiometry of lytic RTX pores remains unknown. In studies with cells, either erythrocytes or other nucleated cells, the dose–response analyses indicate that the lytic activity of the RTX toxins is a highly cooperative function of toxin concentration, suggesting that oligomerization is involved in RTX toxin pore formation [56,57,58]. Moreover, in vitro complementation within pairs of individually inactive deletion variants of *E. coli* HlyA or *B. pertussis* CyaA allowed restoration of, at least in part, the hemolytic and cytotoxic activities, suggesting that two or more toxin molecules had aggregated to form a pore [65,66,67]. However, in other investigations *E. coli* HlyA was recovered from target membranes only as a monomer [51,68]. In the case of adenylate cyclase toxin, Votjova-Vodolanova et al. solved by blue native polyacrylamide gel electrophoresis (BN–PAGE), in erythrocytes membranes, protein bands of ≈400 and 470 kDa, which might correspond to dimers of the 200 kDa toxin polypeptide [69].

Another yet unsettled issue is the calcium-dependence of pore formation by the RTX toxins. Ca^2+^ binding to the RTX repeats domain is considered essential for all biological activities of the RTX toxins, from protein folding to secretion [70,71] including binding to target membranes, and hemolysis or permeabilization of liposomes [72,73]. However, it was reported that *E. coli* HlyA does not need calcium in the medium for its activity towards artificial lipid bilayer membranes, even in the case of HlyA produced in calcium-depleted medium [74]. Similarly, it was noted that the pores formed by *Bordetella* adenylate cyclase toxin (ACT) in asolectin lipid bilayers have single-channel conductance independent of calcium [75]. Determination of very similar or identical single channel conductance values both in the presence and absence of calcium led the authors to propose that calcium influenced only the frequency of channel formation but not the conductance of the single channel [75].

An interesting point regarding the RTX pores is that several studies, including our own data, have revealed that the RTX toxin-induced perturbations in membranes depend on a series of factors such as membrane lipid composition, temperature, incubation time, and toxin concentration [76,77], which has led to the idea that rather than being a static process, permeabilization by RTX toxins may be a complex, dynamic process involving membrane remodeling processes, accompanied by transient formation of non-lamellar intermediates in the membrane. In the case of *E. coli* α-hemolysin several possibilities have been postulated to describe the membrane permeabilization by this toxin, such as a “detergent-like mechanism” [78], “leaky patches” [77], or more recently formation of proteolipidic pores [79]. In a similar line, it has been suggested the membrane destabilization effect by *Aggregatibacter actinomycetemcomitans* leukotoxin LtxA has similar effects [80]. Our last study with the adenylate cyclase toxin [73], which will be discussed below, supports this view that pore formation by the RTX toxins involves lipids.

#### 2.1.2. Adenylate Cyclase Toxin from *Bordetella pertussis*

Adenylate cyclase toxin (ACT or CyaA), a single polypeptide of ≈200 kDa, is crucial for colonization of the respiratory tract and establishment of the disease by *Bordetella pertussis*, the bacterium causative of whooping cough [81,82]. Originally discovered by Hewlett and Wolff [83] in *B. pertussis* culture supernatants, the adenylate cyclase toxin was later found to be activated by the eukaryotic calmodulin [84]. Later on it was shown that adenylate cyclase toxin can enter into eukaryotic cells where, upon activation by calmodulin, it triggers a large increase in cAMP levels [85].

**Figure 3:**
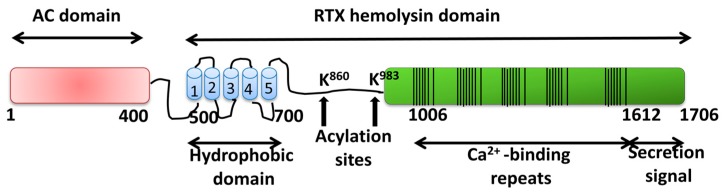
Depiction of the structural organization of the *Bordetella pertussis* adenylate cyclase toxin (ACT) toxin. The catalytic domain (adenylate cyclase (AC) domain) (in red) extends approximately from residues 1 to 400. The repeats in toxin (RTX) hemolysin domain (residues from ≈500 to 1706) contains the hydrophobic domain which is predicted to be constituted by five hydrophobic–amphipathic alpha-helices (1 to 5) (in blue), two conserved acylation sites, K860 and K983, required for activation by palmitoylation mediated by CyaC acyltransferase, and the Ca^2+^-binding region (residues 1006–1612) constituted of five blocks formed by low affinity calcium-binding repeats (in green). In each block multiple lines have been drawn, each line corresponding to single nonapeptide repeats with consensus sequence GGXGXDXLX. The segment between residues 1638–1706 corresponds to the C-terminal secretion signal recognized by the type I secretion machinery.

Synthesis, post-transcriptional modification, and secretion of ACT are determined by the *CyaCABDE* operon [86]. CyaA or ACT, a 1706 amino acid-long polypeptide, is synthesized in the bacterial cytosol as inactive precursor (pro-CyaA or pro-ACT). The pro-toxin is then post-transciptionally acylated at two internal lysine residues (Lys-863 and Lys-913) by a dedicated acyltransferase CyaC, converting into the active toxin form [87]. ACT is then secreted across both bacterial membranes by the type I secretion system composed of CyaB, CyaD, and CyaE proteins. ACT is unique within the pore-forming RTX family of toxins by possessing a cell-invasive N-terminal adenylate cyclase (AC) domain (∼364 residues) fused to a C-terminal RTX hemolysin moiety [86]. The catalytic AC domain converts ATP into cAMP [83,84,88]. The C-terminal RTX moiety (∼1336 carboxy-proximal residues) is responsible for the translocation of the AC domain across the host plasma membrane and for the hemolytic/pore-forming phenotype of ACT. This RTX moiety further consists of: a translocation region, spanning residues ≈365 to 500, which has been directly involved in the transport of the AC domain across the plasma membrane [89], a hydrophobic domain, spanning residues ≈500 to 700, containing several hydrophobic/amphipathic α-helical segments, which has been involved in pore formation by ACT, an acylation region spanning residues 750 to 1000 that contains the two acylation sites (Lys 863 and Lys 913) [87], a calcium-binding RTX domain, between residues 1008 and 1590, which harbors the characteristic Gly- and Asp-rich nonapeptide tandem repeats that form the numerous (∼40) calcium-binding sites of ACT, structured in five blocks, hallmark of ACT membership to the RTX family [90] (see Figure 3), and a C-terminal secretion signal. All ACT biological activities strictly depend on physiological (>0.3 mM) concentrations of free calcium ions in order to fold into an active toxin [91,92].

ACT targets primarily myeloid phagocytic cells that possess the CD11b/CD18 integrin, which acts as toxin receptor [34], although it can also efficiently intoxicate a variety of cells lacking the integrin, such as erythrocytes or epithelial cells, likely through a direct interaction with plasma membrane lipids [86]. To generate cAMP inside the target cell, ACT binds to the cell membrane and translocates directly its AC domain across the plasma membrane by a unique mechanism that does not require endocytosis steps and remains largely undisclosed. Once in the cytosol, the AC domain catalyzes the unregulated conversion of intracellular ATP to cAMP [86]. Besides that, ACT can form pores in membranes that account for the hemolytic activity of the toxin on erythrocytes [56,57]. It is currently believed that the cytotoxic effects of ACT on target cells are consequence of toxin activities, the generation of high cAMP amounts, and the osmotic imbalance caused by its pore-forming activity, which together might contribute to debilitate the host defenses [93,94].

##### Pore-Forming Activity of ACT

By analogy with known pore-forming toxins it may be assumed that the process that ultimately leads to osmotic cell lysis by ACT pores consists of several stages, namely, binding to the membrane (mediated by a specific receptor, or not), insertion into the lipid bilayer, and assembly/oligomerization into a pore structure. Beyond this, our knowledge on the pore-forming activity and the characteristics of the pores formed by ACT is rather limited, and many questions such as the real stoichiometry of the ACT pores, the mechanisms of pore assembly, etc., have remained without definitive answers for almost three decades.

As compared to the rest of toxins of the RTX family the idea has prevailed that ACT is a weak hemolysin and that this low lytic potency is directly related to the very small size of the ACT toxin pores. This concept comes from the first hemolysis assays with osmotic protectants which determined that small sugars such as sucrose (75 mM) protected the erythrocytes from lysis by ACT [56], and from experiments in planar lipid membranes in which the single channel conductance values determined for ACT pores were inferior in more than one order of magnitude relative to those determined for other RTX toxins such as *E. coli* hemolysin (27 pS for ACT at 1M KCl vs. 500 pS for HlyA at 0.15 M KCl) [64]. These data led to the estimation of a diameter of ≈0.6–0.8 nm for ACT pores, far from the estimated ≈2–3 nm of the larger pores formed by several RTX toxins (*E. coli* HlyA hemolysin, ApxI from *A. Pleuropneumoniae*; EHEC-Hly *E. coli* EHEC) using the same type of experiments [50,60]. First deletion mutations in the toxin’s hemolysin domain, which showed a drastic reduction or loss of pore-forming activity of ACT on sheep erythrocytes and artificial bilayer membranes, led to the idea that the toxin portion extending from amino acids 500 to 700 is involved in pore-formation [43,64]. Later prediction studies of the secondary structure of this ACT region defined in ACT sequence five segments with potential amphiphatic or hydrophobic helical profiles that might insert into the lipid bilayer (helix I_502–522_, helix II_529–550_, helix III_571–593_, helix IV_607–627_, and helix V_678–698_) [95,96]. Several mutational studies showed that point mutations in residues located in the putative helixes I and III affect not only pore formation or pore properties, but also AC domain translocation [42]. For example replacement of Glu-509 and Glu-516 in helix I by positively charged lysine residues (E509K and E516K) reduces the capacity of the toxin to translocate the AC domain across membrane and increases the propensity of ACT to form pores, and proline (E516P) or glutamine (E516Q) substitutions extend the lifetime of open single pore units and all three substitutions cause a drop of pore selectivity for cations [42]. A double mutation E509K + E516K further exacerbates the hemolytic and channel forming activity of ACT [95]. Another pair of glutamate residues, E570 and E581 in helix III, has also been shown to affect ACT pore-forming activity. E570P, E570K, and E581P substitutions down-modulate the specific hemolytic activity of ACT, while E581K substitution enhances four times the hemolytic activity of ACT, increasing both the frequency of formation and lifetime of toxin pores. Negative charge at position 570, but not at position 581, was found to be essential for cation selectivity of the pore, suggesting a role of Glu-570 in ion filtering inside or close to the pore mouth [42]. More recently it has been reported that certain mutations in the putative helix II (pro substitution of G531) affect the hemolytic capacity of ACT, but without affecting the AC translocating activity, whereas others (replacement of A538 or A546 by diverse residues) selectively impair the capacity to translocate the AC domain across the cell membrane, without affecting the hemolytic capacity, though strikingly the membrane activity of these mutant toxins in planar asolectin bilayers was very low. It was also shown that the substitution of A538 by a proline residue abolishes the voltage-activated increase of membrane activity of ACT in asolectin bilayers [97]. In other study it was proposed that three Gly residues in helix II (Gly-530, Gly-533, and Gly-537) might be a crucial component of the ACT pore structure and have a role in ACT oligomerization [98].

From all these mutational studies at least two ideas can be drawn, the first one is that the so-called hydrophobic–amphipathic region extending approximately from amino acids 500 to 700 seems indeed to be involved in membrane penetration and pore formation by ACT. The second is that AC domain translocation seems to rely also on the same structural elements (α-helices) involved in pore formation. So, very simplistically and by analogy with the AB_5_ toxins such as diphtheria toxin that translocate the A protein through a pore formed in the membrane by oligomer formed by the B subunit, it could be speculated that translocation of the AC domain could similarly take place through the ACT pore. However, from these and other studies showing dissociating effects of certain residue substitutions in the predicted amphipathic α-helices in the hydrophobic domain affecting selectively only one of the two ACT toxin activities (hemolysis or translocation), a model was postulated in which pore formation and AC translocation are two independent processes that are accomplished in parallel by two distinct conformers of ACT, one accounting for AC domain translocation, the other for formation of membrane pores [42,95,97,99,100]. However it cannot be ruled out that the differential effects of substitutions of single amino acids may be due to that such residues do not form part of the same structural element as has been predicted. In the absence of any structural information on the pore-forming domain of ACT any reliable prediction of its overall organization and the delimitation and topology of its transmembrane structural elements remains difficult.

Interestingly, binding of the monoclonal antibody (MAb) 3D1, which recognizes an epitope (amino acids 373 to 399) at the distal end of the AC catalytic domain, to full length ACT has been shown to prevent the delivery of the catalytic domain to the cytosol of target erythrocytes (with no effect on the enzymatic activity of the toxin (conversion of ATP into cAMP in a cell-free system)) and causes in parallel a three-to fourfold increase in hemolysis by ACT [99]. A deletion mutant (ACT mutant ∆N489) in which the catalytic domain, along with additional amino acids distal to it, was eliminated yielded a similar “hyperhemolytic” phenotype which together has led to postulation that prevention of delivery of the catalytic domain or deletion of the catalytic domain, along with additional amino acids distal to it, elicits a toxin conformation that is more favorable for hemolysis. In the same line, formation of bigger pores combined with higher frequency of pore formation leading to hyperhemolytic phenotype was recently described after substitutions of negatively charged residues located in the AC-to-Hly linker segment of ACT [101]. This and the mutational study with substitutions in amino acids Gly-537, Ala-538, and Ala-546 led to postulate that the AC-to-Hly linker segment together with the structure containing those residues may interact together and control the formation of ACT pores with restricted pore size [97,101].

One early consensus regarding the membranolytic activity of ACT on cells was that the ACT pore likely arises by insertion and assembly of the ACT monomers into higher order oligomers. This conclusion was based on the findings that hemolysis by ACT is a highly cooperative event and complementation assays with pairs of inactive mutants that restored the hemolytic activity [57,65,67]. What it has not yet settled is the possible stoichiometry of such putative ACT oligomers. Using BN–PAGE Votjova-Vodolanova et al. solved in erythrocyte membrane proteins bands of ≈400 and 470 kDa, which might correspond to dimers of the 200 kDa toxin polypeptide [69], while previously Gray et al. had determined a Hill coefficient of ≥3 in hemolysis assays with sheep erythrocytes, which suggests that three or more toxin monomers might be required to form a lytic pore [57,102]. As we will discuss below, ACT might not form a discrete fixed-size pore as has been thought.

Another characteristic of hemolysis by ACT is that relative to the onset of intoxication of red blood cells (AC translocation and cAMP production) which is rapid and maximal in 30–60 min at toxin concentrations of about 1 μg/mL [56], the time course of hemolysis is prolonged (hours), with a lag of >1 h even at toxin concentrations of 10 μg/mL [56]. Interestingly, in the study by Gray et al. [102] it was observed that ACT elicited a rapid increase in K^+^ efflux from cells, and that toxin monomers were sufficient to elicit such K^+^ efflux. The authors suggested in that paper that the transmembrane pathway by which K^+^ is released might be related to, or be a precursor of, that which is ultimately responsible for hemolysis. The hydrodynamic radius of K^+^ ion is ≈0.315 nm or even greater, depending on the method used in the size determination [103], so it would require a pore with a diameter of at least ≈0.63 nm to allow K^+^ diffusion through it. From these data the doubt arises of whether the size of ≈0.6–0.8 nm previously estimated by osmotic protection assays [56] or planar lipid measurements [64] really corresponds to an oligomeric ACT pore, or whether it may be the size of a precursor monomeric ACT pore. It might be speculated that possible oligomeric ACT pores might be larger than 0.6–0.8 nm in diameter. This has been found in our laboratory very recently [73] and will be discussed below.

##### First Nanoscale Pictures of ACT Lytic Pores in Membranes

Very recently we found that the size of the ACT pore is tunable and depends on toxin concentration and on incubation time, and can evolve into a large “hole” of several nm wide, so as to allow the efflux of even small proteins from the target cytosol. Moreover, we have provided the first nanoscale pictures of ACT lytic pores in membranes [73].

**Figure 4:**
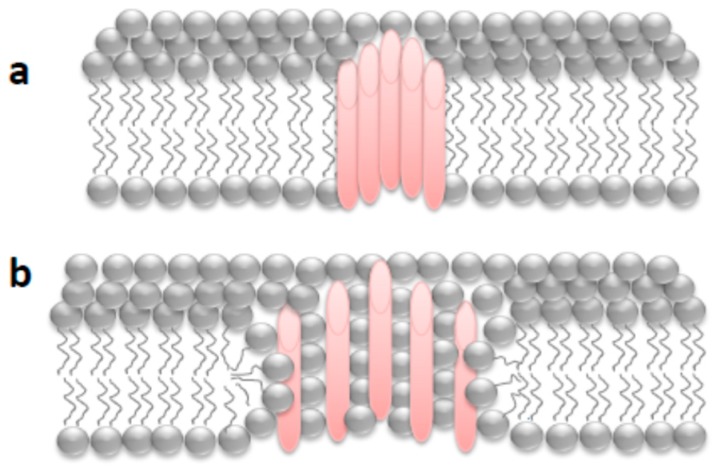
Schematic depiction of different pores with a defined boundary inserted into a biological phospholipid membrane: (**a**) pure proteinaceous pore in which the pore lumen is formed exclusively by protein segments; (**b**) proteolipidic or toroidal pore in which the pore lumen is formed by both lipids and protein segments.

We found that ACT-induced openings in membranes, rather than being fixed-size pores, may be formed through a lipidic mechanism. It is very likely ACT pores are toroidal (proteolipidic) pores, in which both phospholipid molecules and protein segments (likely helical and amphipathic) contribute to delineation of the pore walls (Figure 4). ACT pores thus resemble several amphipathic antimicrobial peptides, or certain pore-forming proteins such as pro-apoptotic Bax that can also be regulated by the lipid:protein molar ratio and has been reported to form toroidal pores in target membranes [104,105,106]. Interestingly, several years ago Welch noted for another RTX toxin, the *Escherichia coli* hemolysin, that the toxin lesion increased in size with time, temperature, and toxin concentration [77] and later another group also noted that *E. coli* hemolysin may form proteolipidic pores [79]. It is thus tempting to speculate that formation of proteolipidic pores might be other common property shared by toxins from the RTX family.

**Figure 5:**
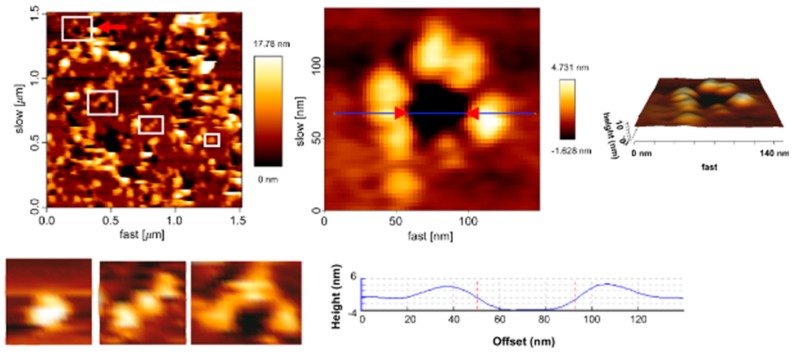
Atomic force microscopy (AFM) picture taken from supported lipid bilayers containing ACT molecules. Analysis by atomic force microscopy of the assemblies formed by ACT in supported lipid membranes. AFM image (left upper panel) of a supported lipid bilayer (SLB) prepared from ACT-containing proteoliposomes palmitoyloleylphosphatidylcholine (POPC liposomes reconstituted with ACT). The red arrowhead points to a membrane pore that has been selected for a more detailed topographic analysis; in the image there are more pores, heterogeneous in size and shape; the edges of the pores present protrusions corresponding to ACT clusters. Below this image, other ACT assemblies are shown, such as a monomer, a line, and an arc, which have been selected from other AFM images. On the right-hand part of the panel, a 3D AFM topography of the pointed ACT ring is shown in a greater detail. The image reveals a circular dark hole that spans the lipid membrane (below). ACT molecules around the pore rim (yellow-white) protrude 3.97 ± 1.02 nm above the membrane plane, as confirmed by the height profile shown below the image (corresponding to the blue line in the middle of the 2D image). Figure taken from Gonzalez-Bullón et al., 2019 [73].

In the mentioned study we obtained atomic force microscopy (AFM) images of ACT lytic pores in membranes. Those pictures showed that ACT forms assemblies of heterogeneous architectures, in the form of lines, arcs, and closed rings, inserted in pure lipid bilayers made of phosphatidylcholine, and that the ACT arcs and rings pierce the membrane, corresponding thus to real ACT lytic pores. We found ACT “holes” with wide and variable sizes, some of which showed an internal diameter of about 20 nm in the narrowest part of the vestibule and ≈50–60 nm in the more external part limiting the aqueous medium (Figure 5). Moreover, using cell-sized giant unilamellar vesicles (GUVs) we observed an efficient filling of the vesicles with fluorescent dextrans of large molecular masses ( FITC-Dextran 4 kDa Stokes radius of 1.4 nm, FITC-Dextran 10 kDa Stokes radius of 2.36 nm, FITC-Dextran 20 kDa Stokes radius of 3.30 nm) [73], which was consistent with the AFM data and affirmed that ACT can form large membrane pores, of several nanometers wide, much greater than previously reported [56,64]. Pores as large as these had indeed only been reported for cholesterol-dependent cytolysins, such as listeriolysin O or streptolysin O, that create β-structured membrane pores.

We were also able to resolve by BN–PAGE, both in lipid vesicles and in cells (macrophages and CR3-negative cells) under lytic conditions, protein bands with apparent molecular masses of ≈550, 800, 1000, and 1200 kDa, which may correspond to ACT trimers, tetramers, pentamers, and hexamers [73] The data were thus in full agreement with previous studies showing that hemolysis by ACT is a highly cooperative event with Hill number ≥3 [57]. Previously another group had solved by BN–PAGE smaller ACT oligomers of apparent molecular masses of ≈410 and 470 kDa in toxin-treated erythrocytes, corresponding perhaps to ACT dimers [69]. In those experiments sheep erythrocytes were treated with ACT for 30 min and since for detecting hemolysis incubations of ≈180–240 min are usually used, it can be speculated that such smaller assemblies might grow into larger ACT oligomers, similar to the ones detected in our present study, at longer toxin treatments. There is another early study on hemolysis by ACT [56] in which the authors concluded that the ACT pore size has to be ≤0.62 nm, since they observed that small sugars such as arabinose (75 mM) (estimated diameter ≈0.62 nm) protected red blood cells from hemolysis [56]. Our study has revealed however that ACT may form large holes of variable effective diameters of several nm in membranes. The question is whether these results are mutually exclusive. We think that the growing size model we have proposed for ACT pores may explain and reconcile the seemingly contrasting results, and so, it can be expected that large ACT pores would form whenever enough toxin concentrations and incubation times are used so as to allow toxin oligomerization. In the mentioned osmotic protection study [56], red blood cells (RBC) were supposedly incubated with ACT for a very short time (15 min) and then washed to continue with incubation for 5 h in presence of the protectants [56]. A short exposure time and a washing step might have left less toxin molecules at the cell membrane, hindering formation of large oligomers, and so only small (monomeric?) pores might have formed, which would be protectable by small sugars.

Our AFM pictures also show that besides the large toxin assemblies, abundant free ACT monomers inserted into the lipid membrane coexist. Both the ACT assemblies (lines, arcs, and rings) as well as the monomers detected have great height variability in their constituent monomeric particles, with height values from ≈3.0–6.0 nm for the monomeric particles in lines and arcs, to values of ≈2.0–3.0 nm for the closed rings. Interestingly, the free ACT monomers show the greatest height variability, protruding between ≈3.0 to 8.0 nm from the plane of the lipid bilayer. We hypothesize that these differences in the monomer height reflect different penetration degrees of the ACT monomeric particles into the lipid bilayer, and it appears that a correlation exists between the monomer insertion degree and the assembly order (ring > arc > line > monomer), suggesting that the deeper the ACT “monomeric particle” inserts, the larger the oligomer that can be formed. All these data has led us to postulate that pore formation by ACT might follow the so-called “non-concerted pathway” [11,107] in which the water-soluble monomers bind first to the membrane in a likely fast step, and then membrane insertion of the monomer may take place earlier than (or concomitantly with) oligomerization, as detection of intermediate pore stages with lower stoichiometries is possible [11,107]. A non-concerted insertion model was previously proposed for pores formed by the proapoptotic Bax protein, or by toxins of the actinoporin family that insert α-helices into the membrane [11,107]. This model of pore formation is associated with pores whose walls are lined both by lipids and proteins and it is characteristic of heterogeneous and flexible architectures [11]. The AFM data were thus consistent with the permeabilization data in GUVs, and reinforce the idea that ACT forms in membrane proteolipidic toroidal pores.

Recently Canella et al. [108] have provided the first in solution structural characterization of the calcium-loaded monomeric ACT, which shows that the full length toxin adopts in solution a compact and stable state with a mean diameter of ≈14–15 nm, in which the toxin’s calcium-binding C-terminal domain stabilizes the N-terminal catalytic domain [108]. Strikingly, the (total) diameter of the ACT monomer on the membrane as determined in our AFM study, ranges from the ≈60 nm (free monomer), to the ≈50 nm (monomer in the lines and arcs), and to ≈40 nm (closed rings) (Figure 5), suggesting that upon binding to the membrane, the ACT polypeptide would undergo a (rapid?) transition from a compact state in solution, to an open, extended state on the lipid surface. The initial binding and opening of the ACT monomer would be likely followed by protein–lipid interactions and subsequent reorganization of the ACT polypeptide leading to a progressively more inserted state (height of the monomeric particle in monomers goes from ≈8.0 nm to ≈3.0 nm, from the plane of the membrane) and this progressive insertion of the monomer chain, which is parallel to a decrease in particle diameter, might bring the particles closer to each other, thus favoring protein–protein interactions and promoting ACT oligomerization (height values of the monomeric particle in the closed rings are ≈2.0–3.0 nm) and formation of larger lytic pores. The observed ACT lines and arcs (height values between ≈3.0–6.0 nm) might be, in this context, “intermediates” of oligomerization, supporting a “sequential” mode of oligomerization [11], which involves the successive addition of units with the same stoichiometry (likely ACT monomers). This model has been described for proteins forming toroidal proteolipidic pores in membranes [11]. From our permeabilization and AFM data we hypothesize that the kinetics of ACT oligomerization may be directly conditioned by the kinetics of penetration into the lipid bilayer of the toxin monomers, perhaps this step being the rate limiting one for the assembly of oligomeric ACT lytic pores.

## 3. Conclusions

After more than thirty years our knowledge on the pore structure that ACT or other RTX toxins create in the plasma membrane to induce target cell permeabilization remains very limited, and though the abundant mutagenesis studies have resulted in valuable tools to define segments important for pore-forming activity, we still lack a precise delineation of the structural elements directly involved in ACT toxin pores. The early determination of very low single-channel conductance values for a presumably oligomeric pore formed by ACT in lipid bilayers has strongly conditioned the field for long time, but interestingly the most recent data revealing that ACT-induced openings in membranes, rather than being static fixed-sized small pure proteinaceous pores, may be large, heterogeneous, and formed through a lipidic mechanism has opened a new window to understand pore formation by ACT and perhaps by other RTX toxins. Relative to the progress made with other pore-forming toxins and proteins the RTX field is in its infancy, but instead of being an obstacle, this deficit should encourage different laboratories in the field to continue working hard to unveil, one day, the secrets of the RTX toxin family. In this review we have included descriptions of proteolipidic pores of tunable size formed by a bacterial pore-forming toxin. This result might be very inspirational for scientists looking to adjust the physical properties of the pores for various sensing purposes.
